# Icariin Prevents Extracellular Matrix Accumulation and Ameliorates Experimental Diabetic Kidney Disease by Inhibiting Oxidative Stress via GPER Mediated p62-Dependent Keap1 Degradation and Nrf2 Activation

**DOI:** 10.3389/fcell.2020.00559

**Published:** 2020-07-17

**Authors:** Kai Wang, Xiulan Zheng, Zhenzhen Pan, Wenhui Yao, Xin Gao, Xiniao Wang, Xuansheng Ding

**Affiliations:** School of Basic Medicine and Clinical Pharmacy, China Pharmaceutical University, Nanjing, China

**Keywords:** diabetic kidney disease, icariin, oxidative stress, extracellular matrix, GPER

## Abstract

The present study aimed to determine whether icariin could attenuate type 1 diabetic nephropathy (T1DN) induced by streptozotocin (STZ) after 4 weeks or not. Therefore, its therapeutic effect on diabetic kidney disease was investigated in view of reactive oxygen (ROS) and extracellular matrix (ECM) generation in human glomerular mesangial cells under high glucose. To establish the participation and the key role of GPER and Nrf2 in ECM deposition, a combination of G15 (antagonist of GPER) or siGPER and siNrf2 were performed, respectively. The results showed that T1DN can be significantly inhibited by oral icariin, evidenced by improvement of 24 h urinary volume, 24 h proteinuria, microalbuminuria, and histopathological changes of kidney. Icariin decreased the levels of intracellular superoxide anion, impeded the generation of fibronectin and increased the expression and activity of antioxidant enzymes in the human glomerular mesangial cells treated with high glucose. It acted as a GPER activator, increased dissociation of Nrf2/Keap1 complexes, combination of Keap1/p62 complexes, Nrf2 translocation to nuclear, Nrf2/ARE DNA binding activity, and ARE luciferase reporter gene activity in glomerular mesangial cells. The Nrf2 inhibitor ML385 or siNrf2 obviously abolished extracellular matrix (ECM) generation inhibited by icariin. Furthermore, icariin-induced Nrf2 activation was mainly dependent on p62-mediated Keap1 degradation, which functions as an adaptor protein during autophagy. The GPER antagonist G15 and siGPER obviously abolished the above effects by icariin. Taken together, the present study demonstrated that the therapeutic effects of icariin on type 1 diabetic nephropathy in rats via GPER mediated p62-dependent Keap1 degradation and Nrf2 activation.

## Introduction

Diabetic nephropathy (DN), a common devastating complication of both types of diabetes and supervenes as the result of microvascular lesions in the renal glomeruli, is often associated with increased cardiovascular mortality and reduced life quality. According to previous studies, about 30% of patients with type 1 diabetes (T1D) and 20% of patients with type 2 diabetes (T2D) would ultimately be followed up with diabetic nephropathy. Approximately 25% of T1D occurred microalbuminuria after 15–20 years. By reporting, the end-stage renal disease incidence can be 4–17% within 25 years after the diagnoses of T1D. Over 20% of patients with T2D already have diabetic nephropathy at the same time of diabetes (Parving et al., 1988), of whom a further 35% develop DN mostly in next 10 years. Diabetic nephropathy, known as glomerulosclerosis, is associated with progressive, uninterrupted scarring of the glomerulus (Wolf and Ziyadeh, 1999; Zheng et al., 2004). Diabetic induce oxidative stress and excessive deposition of extracellular matrix (ECM) in the mesangium of the glomerulus (Mason and Wahab, 2003). The phenomenon is attributed to the loss of glomerular mesangial cell viability, which is a crucial-pathological change in the diabetes-mediated renal injury (Ortiz-Munoz et al., 2010; Loeffler et al., 2011).

Increasing evidence suggests the oxidative stress as a common feature connecting the changes of metabolic pathways in kidneys (Munoz et al., 2020). Renal oxidative stress is initiated by alterations of advanced glycation end products and glucose metabolism, leading to the antioxidant depletion and ROS production induced by pro-oxidant enzyme increasing (Jha et al., 2016). The investigation on regulation of the transcription factor Nrf2 has always been active. During the resting state, Nrf2 was regulated by Keap1 and kept its level low by ubiquitination and proteosomal degradation in cytoplasm (Loboda et al., 2016). In the case of stimulation, Nrf2 would induce the expression of protective, so-called phase 2 antioxidant genes, such as NQO1, HO-1, SOD2, and Trx1 by interacting with anti-oxidative response elements in the promoters for the maintenance of redox homeostasis (Rueda et al., 2007).

**Figure 1:**
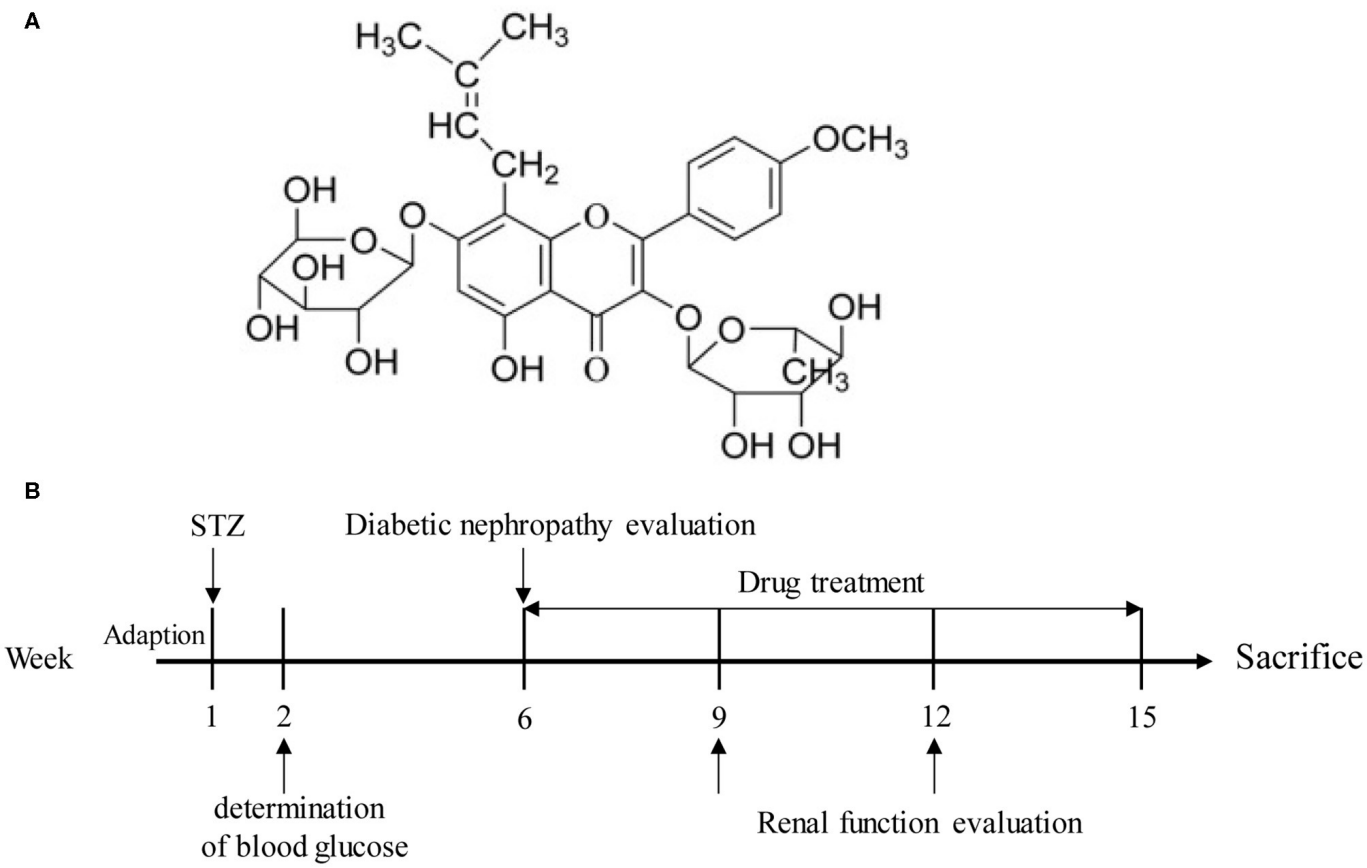
**(A)** Structure of icariin. **(B)** A schematic representation of the diabetic nephropathy procedure and treatments in rats.

Icariin (Figure 1A) is the flavonoids used to remedy the kidney disease in traditional Chinese medicine, which is extracted from Epimedium species. Scientific research has found that icariin possesses multiple bioactivities such as protecting neurons from injury, improving osteoporosis, anti-inflammation, anti-tumor, anti-depression, and anti-oxidation functions (Yao et al., 2019). Recent studies have confirmed that icariin exerted preventive function of kidney disease in streptozotocin (STZ)-induced diabetic rats (Qi et al., 2011). In present, we investigated the potential of icariin in the treatment of DN and explored its mechanism of action from the perspective of ROS production in glomerular mesangial cells.

## Materials and Methods

### Chemicals and Regents

Icariin was obtained from Yangtze River Pharm Co., Ltd. (Taizhou, China). Irbesartan was obtained from Sanofi San de la Fort Group Co., Ltd. (Paris, France). Streptozocin (STZ) was purchased from Sigma-Aldrich (St. Louis, MO, USA). Urine protein test kit, total cholesterol assay kit, low-density lipoprotein cholesterol assay kit, high-density lipoprotein cholesterol assay kit, triglyceride assay kit, creatinine assay kit, Heme oxygenase 1 (HO1), Quinone oxidoreductase 1 (NQO1) enzyme activity detection kits and malondialdehyde (MDA) were gained from Nanjing JianCheng Bioengineering Institute (Nanjing, China). Rat insulin ELISA kit was obtained from Jiangsu Yutong Biotechnology Co., Ltd (Nanjing, China). Blood glucose meter and Blood glucose test strips were obtained from Roche (Basel, Switzerland). Nuclear and cytoplasmic protein extraction kit and Phenylmethanesulfonyl fluoride (PMSF) were obtained from KeyGen Biotech (Nanjing, China). TRIzol reagent was purchased from Invitrogen (California, USA). DAPI, SOD, GPx, CAT enzyme activity detection kits and the goat anti-rabbit secondary antibodies were obtained from Beyotime Biotechnology (Shanghai, China). PI3K, Akt and mTOR antibodies were obtained from Cell Signaling Technology, Inc (Massachusetts, USA). ML385 was obtained from MCE (New Jersey, USA). Heme oxygenase 1 (HO1), superoxide dismutase 2 (SOD2) /MnSOD, and thioredoxin 1 (Trx1) antibodies were purchased from ProteinTech (Chicago, USA). Fibronectin, NQO1 and Nrf2 antibodies were purchased from Abcam (Chicago, USA). p62 antibody was obtained from Wanleibio (Shenyang, China). G15 was purchased from ApexBio (Houston, USA). Fetal bovine serum (FBS) was purchased from Biological Industries (BI, Israel). GAPDH and β-actin antibodies were purchased from Abways Technology (beijing, China). Dihydroethidium (DHE) was purchased from Yeasen (Shanghai, China). TRIzol reagent, qPCR SYBR® Green Master Mix and Enhanced chemiluminescent (ECL) plus reagent kit were purchased from Vazyme (Nanjing, China).

### Animal Experiments

Female Spraque–Dawley rats (weighing between 180 to 200 g, *n* = 104) were obtained from Zhejiang Experimental Animal Center. Throughout experimental process, rats were housed in a controlled environment condition. The experimental protocol was reviewed and approved by Institutional Animal Care and Use committee of China Pharmaceutical University. The approved number of animal experiments was 2019-05-16.

For intraperitoneal injection, STZ was dissolved in citrate buffer (PH 4.5) and administered in a dose of 55 mg/kg. After 1 week of STZ administration, the rats with fasting blood glucose level over 11.1 mmol/L and persistent hyperglycemia over 16.7 mmol/L were considered as diabetic. Four weeks later, the serum urea nitrogen, urine protein and creatinine levels of rats were measured, and the rats (*n* = 98) meeting the requirements were selected for experimentation (Figure 1B). Rats were randomly divided into six groups: normal group, model group, icariin (20, 40, 80 mg/kg, i.g.) group and irbesartan (13.5 mg/kg, i.g.) group. After 9 weeks of administration, the blood and urine samples of all the rats were gathered. Finally, the rats were sacrificed, and their kidney tissues were obtained and weighed. For hematoxylin-eosin (H&E), Masson and PAS staining, part of kidney tissues were fixed in 4% paraformaldehyde. The rest of kidney tissues were preserved at −80°C for western blot analysis. The serum of rats was collected and stored for the detection of certain biochemical indexes.

### Immunohistochemistry Assay

For immunohistochemistry assays, kidney tissues were fixed in 4% paraformaldehyde and embedded in paraffin. Then, the renal sections were daparaffinized, subjected to antigen retrieval and treated with 0.5% H_2_O_2_ to eliminate endogenous peroxidase activity. Next, the sections were incubated in blocking buffer (2% BSA) with primary antibody overnight at 4°C. Nrf2 and Fibronectin used at a dilution of 1:200 and 1:100, respectively.

### ELISA Assay

To detect the levels of urea nitrogen and serum creatinine, ELISA assays were performed according to the manufacturer's instructions. All ELISA kits were purchased from Nanjing JianCheng Bioengineering Institute (Nanjing, China).

### Cell Culture

Human mesangial cells (HMC) was obtained from ScienCell Research Laboratories, Inc. (San Diego, California, USA). Cells were cultured in Dulbecco's Modified Eagle's medium (DMEM) containing 10% FBS (fetal bovine serum) and Penicillin-Streptomycin Solution (1X) in an atmosphere of 5% CO_2_ at 37°C. For the experimental study, HMCs were maintained in DMEM with normal glucose (5.6 mM glucose, NG), high glucose (30 mM glucose, HG), icariin of different concentrations and irbesartan of a certain concentration in the presence of high glucose for 48 h.

### Western Blot Assay

The whole and nucleus proteins were lysed by certain lysis buffer containing 1% PMSF on the ice, and then clarified by centrifugation at 4Â°C. The samples were then separated by 10 % SDS-PAGE and electroblotted onto PVDF membranes. The membranes were blocked with 5% non-fat milk in TBST for 1.5 h, and incubated with Nrf2 (1:1,000), SOD2 (1:1,000), NQO1 (1:1,000), HO1 (1:1,000), Trx1 (1:1,000), Fibronectin (1:1,000), β-actin (1:1,000), and GAPDH (1:1,000) at 4°C overnight. Next, the membranes were washed and incubated with goat anti-rabbit IgG (1:5,000) for 1.5 h at room temperature. Finally, using ECL reagent (vazyme) to make the membrane visible and analytical, and all these images were quantified by ImageJ Software (Media Cybernetics, Silver Spring, MD, USA). Data were expressed as the ratio of integrated optical density to area.

### Immunofluorescence Assay

HMC (1 × 10^6^ cells per well) were plated on glass coverslips. The cells were fixed in 4% paraformaldehyde, permeabilized with 0.3% Triton X-100 in PBS for 20 min at room temperature and incubated in blocking buffer (1% BSA) for 1 h. Then, Nrf2 antibody (1:200) was added in the glass coverslips at 4°C overnight. After washing cells with PBS for 3 times, goat anti-rabbit IgG H&L (FITC) was added and incubated for 1 h at room temperature. For nuclear staining, cells were incubated with DAPI (1:100,000) for 10 min. Finally, the images of cells were gathered by fluorescence microscope (Olympus, Japan) in the China Pharmaceutical University.

### Co-immunoprecipitation Assay

HMC were treated with indicated drugs for 48 h, washed with cold phosphate-buffered saline (PBS) three times, and harvested by centrifugation. Then, the nuclear and cytoplasmic fractions were prepared by commercial nuclear and cytoplasmic extraction reagents. Proteins isolated from HMC were incubated with Nrf2 or Keap1 antibody at 4°C overnight. Next, the proteins were precipitated using protein A/G-agarose beads for 4 h at room temperature. After washing the precipitated proteins with PBS for 3 times, SDS-PAGE was used to separate proteins as illustrated in the western blot assay.

### siRNA Transfection

Before transfection, 1 × 10^6^ HMC cells were seeded in 6-well. 24 h later, the cells were transfected with the Control siRNA, siNrf2 or siGPER by lipofectamine 2000 reagent (Invitrogen, Carlsbad, CA, USA) and culture in opti-MEM reduced serum medium for 4 h. Next, the opti-MEM reduced serum transfection medium was removed from plates and DMEM with 10% FBS was added for 36 h incubation. Then, cells were treated by indicated drugs and harvested. Lastly, the samples were subjected to Q-PCR assay or western blot assay according to manufacturer's instruction with some modification.

### Quantitative-Polymerase Chain Reaction (Q-PCR)

Kidneys cortexes from control and diabetic mice were collected or HMC cells were isolated to extract RNA using Trizol followed by the manufacturer's instructions. Total RNA was dissolved in the DEPC water. The RNA concentration was determined with a spectrophotometer (Nano 1000, Thermo Fisher Scientific, Waltham, MA, USA) by absorbance at 260 nm. Then, the reverse transcription of purified total RNA (1 μg) was conducted by HiScript™ reverse transcriptase kit and then reverse transcriptase–polymerase chain reaction was conducted on ABI 7500 (Applied Biosystems, Foster City, CA, USA) with Hieff™ qPCR SYBR® Green Master Mix. GAPDH was used as a control for target gene. The sequences of primers were listed in Tables 1, 2.

**Table 1:** Human Primers used in quantitative-polymerase chain reaction (Q-PCR).

**Gene**		**Sequence (5^**′**^-3^**′**^)**
GAPDH	Forward	TCGTGGAGGGACTTATGA
	Reverse	AGTGAGTGTCGCTGTTGA
GPER	Forward	CGTCATTCCAGACAGCACCGAG
	Reverse	CGAGGAGCCAGAAGCCACATC
Nrf2	Forward	CGGTATGCAACAGGACATTG
	Reverse	TTGGCTTCTGGACTTGGAAC
NQO1	Forward	CGCGACTCCCACAAGGTT
	Reverse	GTCCGACTCCACCACCTCC
Trx1	Forward	CGGAGAGGAGACTTCACAGAG
	Reverse	ATTTCCACGATTTCCCAGAG
SOD2	Forward	CAATAGAAGGCTGCCCTTTC
	Reverse	CACAGTGCACAGGAACACAG
HO-1	Forward	GGCAGAGGGTGATAGAAGAGG
	Reverse	AGCTCCTGCAACTCCTCAAA

**Table 2:** Rat Primers used in quantitative-polymerase chain reaction (Q-PCR).

**Gene**		**Sequence (5^**′**^-3^**′**^)**
GAPDH	Forward	TGAACGGGAAGCTCACTG
	Reverse	GCTTCACCACCTTCTTGATG
NQO1	Forward	GGGGACATGAACGTCATTCTCT
	Reverse	AGTGGTGACTCCTCCCAGACAG
Trx1	Forward	ATGACTGCCAGGATGTTGCT
	Reverse	GTTAGCACCAGAGAACTCCCC
SOD2	Forward	GACGCCGCAGAGCAGAC
	Reverse	GACACAACATTGCTGAGGCG
HO-1	Forward	TGTCCCAGGATTTGTCCGAG
	Reverse	ACTGGGTTCTGCTTGTTTCGCT

### Superoxide Measurement

To detect the superoxide, HMC were treated with irbesartan (1 μM) and icariin (1, 3, 10 μM) for 48 h with or without high glucose. The final concentration of dihydroethidium (DHE) was 10 μM (Yeasen, Shanghai, China) with serum-free medium. Remove the cell culture medium, add appropriate volume of DHE, and incubate in 37°C cell incubator for 30 min. After washing cells with serum-free cell culture solution for 3 times, cells were collected and treated according to the manufacturer's instructions. Lastly, cells were detected by flow cytometry (Macsqant, Germany) in the China Pharmaceutical University.

To detect the superoxide level in the kidney tissues of rats, frozen slides were incubated with 10 μM DHE for 30 min at 37°C in dark. After washing slices with PBS for 3 times, they were photographed by fluorescence microscope.

### Detection of Antioxidant Enzyme Activity

HMC cells (1 × 10^6^ cells per well) were cultured in a 6-well plate at the logarithmic growth stage. The cells were completely adherent to the wall. Added low sugar culture medium to the normal group, high sugar culture medium (30 mM) to the model group, and treat the administration group with corresponding concentration of drugs and high sugar culture medium, respectively. After 48 h of administration, discarded the culture medium, washed the residual complete culture medium twice with PBS, and discarded all the residual PBS as much as possible. The activities of SOD, HO-1, NQO1, GPx and CAT antioxidant enzymes were detected according to the product manual of commodity manufacturer.

### Electrophoretic Mobility Shift Assay (EMSA)

EMSA was performed to detect the DNA binding activity of Nrf2 by a commercial kit (Pierce, Rockford, IL, USA). Briefly, HMC (1 × 10^6^ cells per well) were cultured in 6-well plates and treated with indicated drugs for 48 h and the nuclear proteins were extracted. Then, the proteins were incubated with biotin-labeled Nrf2-specific oligonucleotides, poly (dI-dC) and binding buffer for 20 min at room temperature. Next, the protein-DNA complexes were separated on a 5% non-denatured polyacrylamide gels and transferred onto PVDF membranes. Lastly, the biotin end-labeled DNA was detected by chemiluminescence.

### Luciferase Reporter Assay

For Luciferase reporter assay, HMC were co-transfected with pGL3-ARE reporter gene plasmid by Lipofectamine 2000 for 8 h. Then, fresh medium with indicated drugs were added for another 48 h. Lastly, the relative luciferase activity was detected by the Luciferase assay kit.

### Statistical Analysis

All experiments were performed for at least 3 times and the data was shown as mean ± SD. Data among groups were analyzed by *T*-test (two groups) or one-way ANOVA (multiple groups) by using SPSS statistical software (SPSS, Chicago, IL, USA). *p* < 0.05 was considered as statistically significant.

## Results

### Evaluation of Diabetic Nephropathy Model in STZ-Induced Diabetic Rats Before Administration

**Figure 2:**
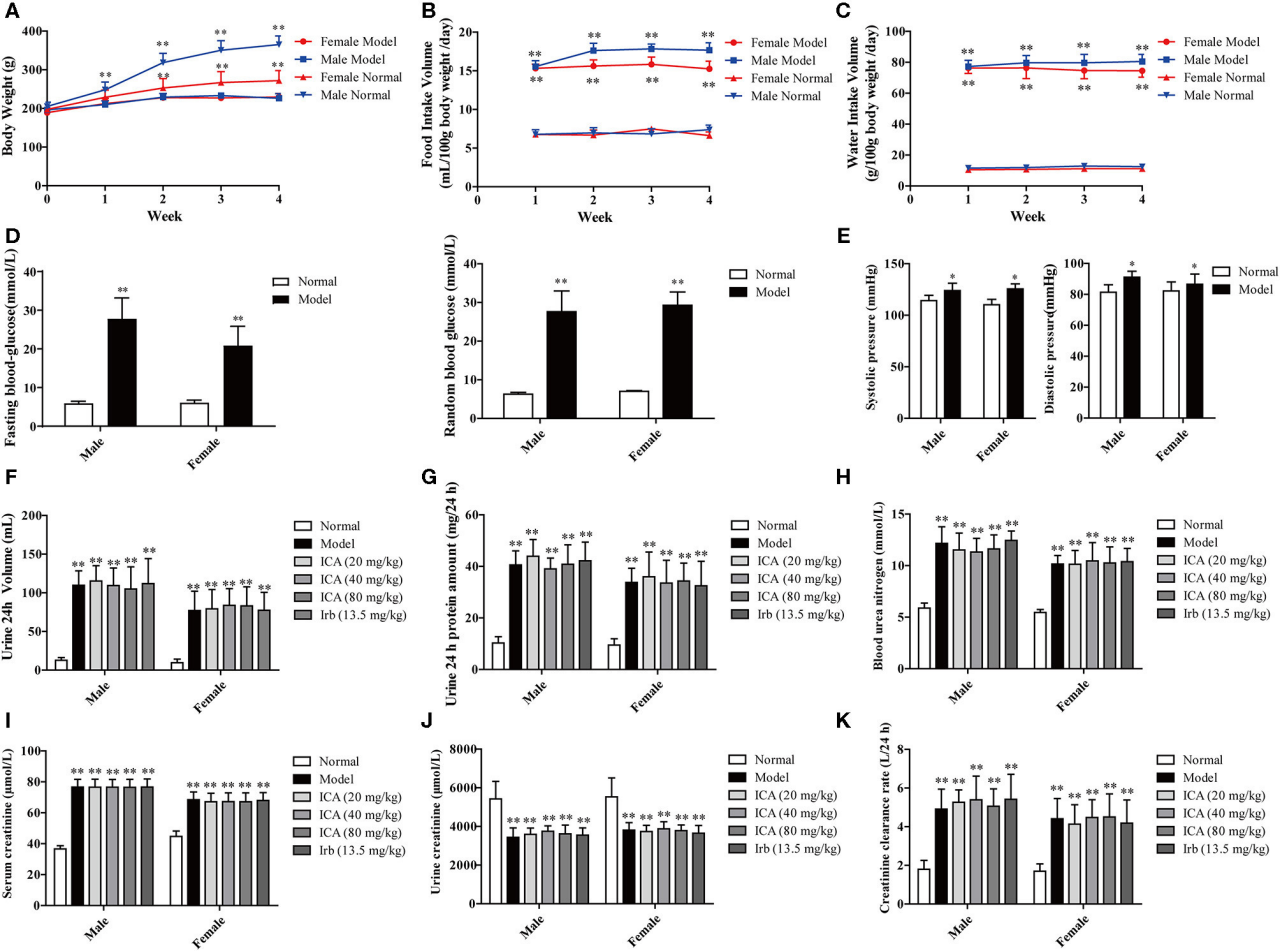
Renal function in STZ-induced diabetic rats before administration. Four weeks after STZ administration, diabetic nephropathy models were examined based on general indicators. **(A)** Weight loss. **(B)** Food intake. **(C)** Water intake. **(D)** Blood glucose. **(E)** Blood pressure. **(F)** 24 h urine volume, **(G)** 24 h proteinuria, **(H)** Blood urea nitrogen (BUN), **(I)** Serum creatinine (Src), and **(J)** Urine creatinine (Ucr) were measured by ELISA. **(K)** Creatinine clearance rate. Data are expressed as mean ± SD. *p < 0.05, **p < 0.01, compared with the normal group.

Four weeks after STZ injection, diabetic rats showed a remarkably increase in food intake (Figure 2B), water intake (Figure 2C), blood glucose (Figure 2D), blood pressure (Figure 2E), 24 h urine volume (Figure 2F), 24 h proteinuria (Figure 2G), blood urea nitrogen (BUN) (Figure 2H), serum creatinine (Src) (Figure 2I), and creatinine clearance rate (Figure 2K) while reduction in urine creatinine (Ucr) (Figure 2J) and weight (Figure 2A) when compared with the normal rats. The result showed that diabetic rats were developed with diabetes nephropathy. Subsequently, healthy rats with the injection of 0.1 M citrate buffer and successful established DN rats were divided into six groups.

### Icariin Prevented the Development of DN in STZ-Induced Diabetic Rats

**Figure 3:**
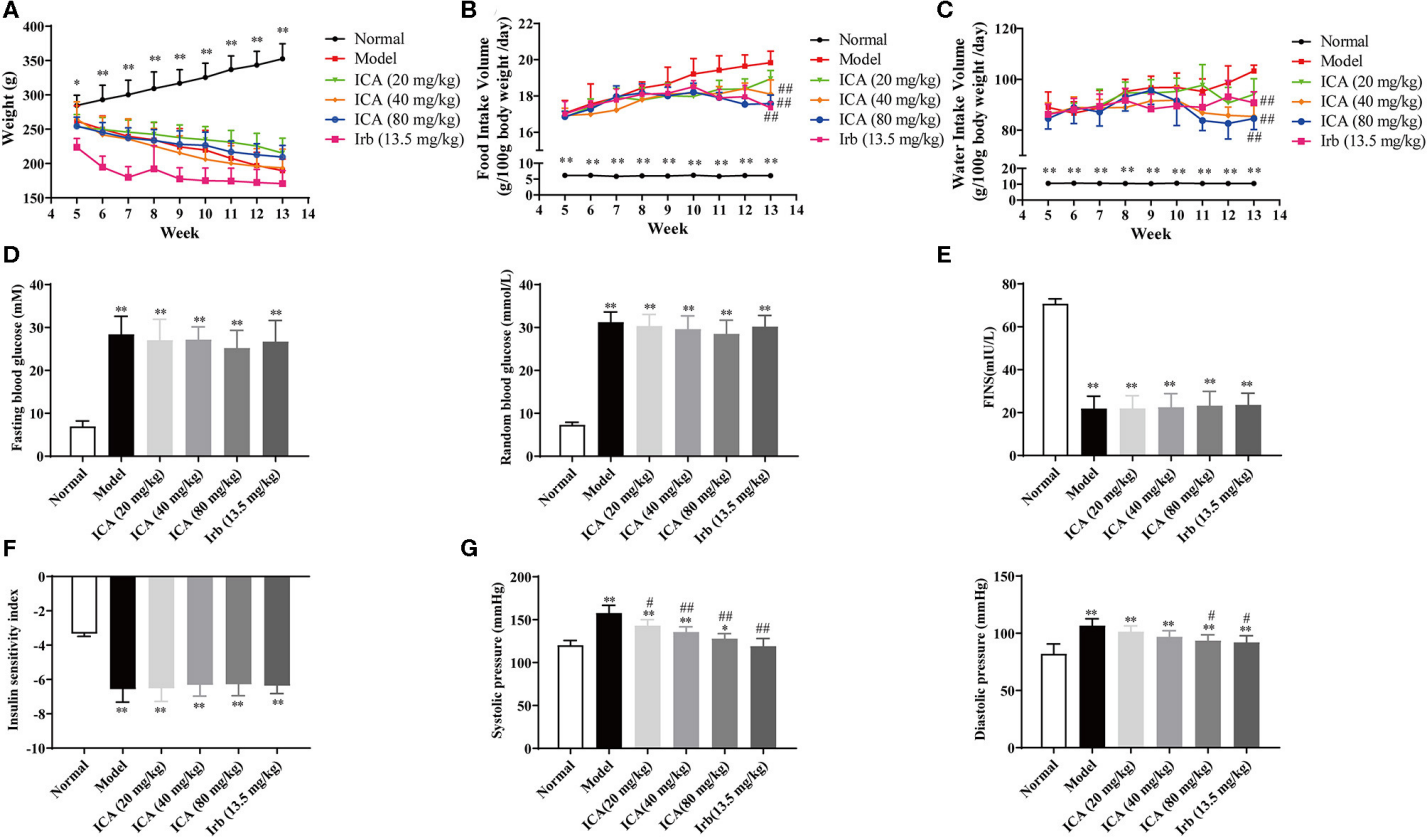
Icariin prevented the development of diabetes in STZ-induced diabetic rats. Rats were subjected to diabetic nephropathy, and orally administered with icariin (20, 40, 80 mg/kg) or irbesartan (13.5 mg/kg) daily for 9 weeks. Rats were sacrificed, and kidneys were isolated. **(A)** Body weight. **(B)** Food intake. **(C)** Water intake. **(D)** Blood glucose. **(E)** Fasting serum insulin. **(F)** Insulin sensitivity index. **(G)** Blood pressure. Data are expressed as mean ± SD. *p < 0.05, **p < 0.01, compared with the normal control group; ^#^*p* < 0.05, ^*##*^*p* < 0.01, compared with the Model group (*n* = 10).

Body weight, food intake and water intake were recorded every day during the 9-weeks period. Diabetes is often associated with weight loss and increased water and food intake. Our results showed that diabetic rats took more water and food, along with the hyperglycemia and high blood pressure, indicating that a DN animal model was successfully established. Interestingly, icariin dose-dependently attenuated the progression of DN, as exhibited by decreased weight loss (Figure 3A) and inhibited the increase in water (Figure 3C), food intake (Figure 3B) and blood pressure (Figure 3G), but no significant changes in blood glucose (Figure 3D), fasting insulin level (Figure 3E) and insulin sensitivity index (Figure 3F) were observed in the different groups, which suggested that the improvement of diabetes symptoms by oral icariin was probably independent of glycemic control.

### Icariin Dose-Dependently Ameliorated Renal Function and Corrected of Dyslipidemia in STZ-Induced Diabetic Rats

**Figure 4:**
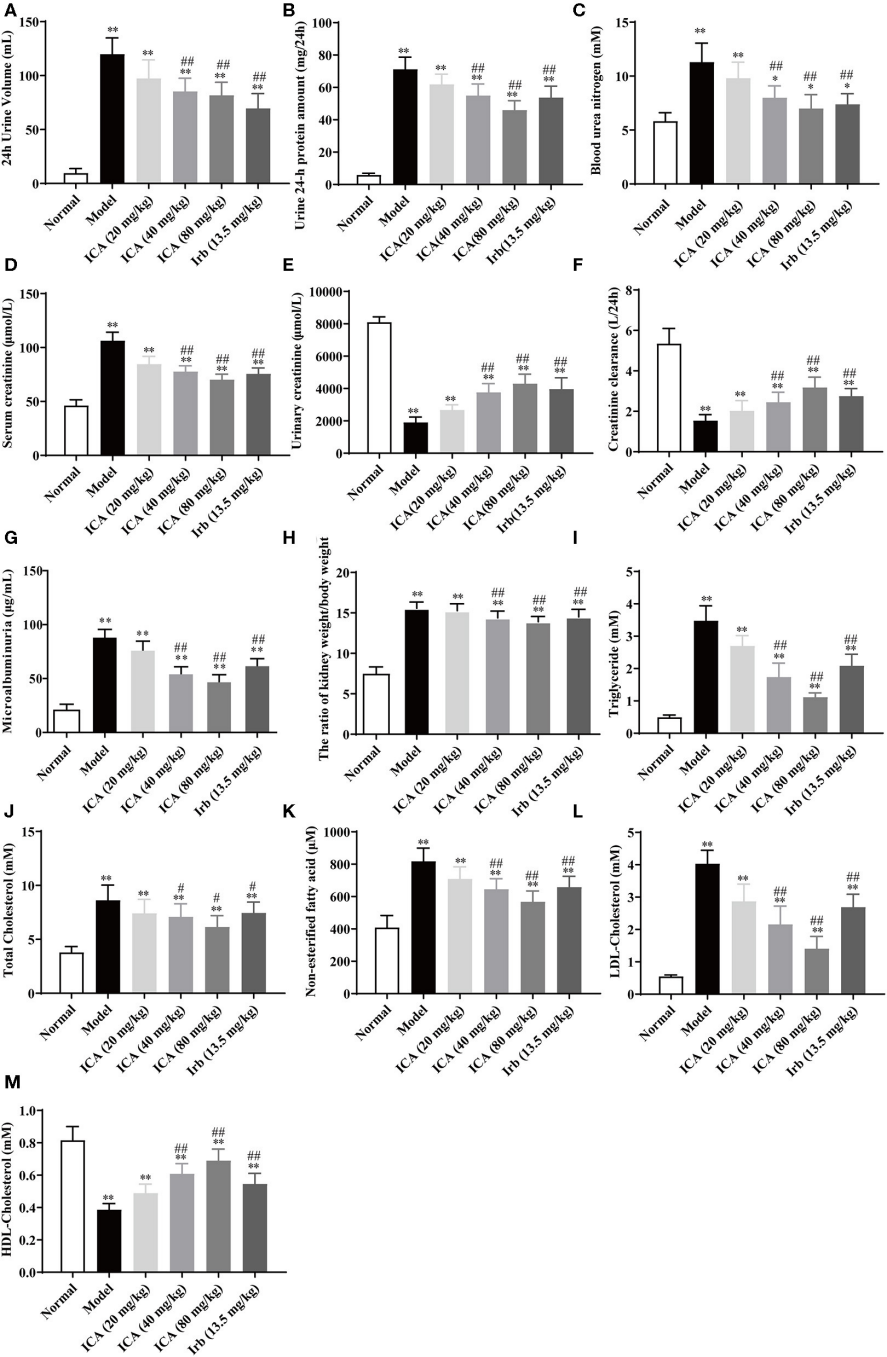
Effects of icariin on metabolic parameters in kidney damage rats. **(A)** The 24 h urinary volume, **(B)** 24 h proteinuria, **(C)** Blood urea nitrogen (BUN), **(D)** Serum creatinine (Src), **(E)** Urinary creatinine, **(F)** Creatinine clearance, and **(G)** 24 h urinary albumin excretion were measured by ELISA. **(H)** The ratio of kidney weight to body weight. **(I)** Triglyceride, **(J)** Total Cholesterol, **(K)** Non-esterified fatty acid, **(L)** LDL-Cholesterol and **(M)** HDL-Cholesterol were measured by ELISA. Data are expressed as mean ± SD (*n* = 10). *p < 0.05, **p < 0.01, compared with the normal control group; ^#^*p* < 0.05, ^*##*^*p* < 0.01, compared with the Model group.

Nine weeks after being grouped, kidneys in diabetes mellitus (DM) rats in model group were heavier than those in other groups (Figure 4H). However, these indicators of kidney function were dose-dependently increased in icariin treatment groups of diabetic rats. The 24 h urinary volume (Figure 4A), 24 h proteinuria (Figure 4B), blood urea nitrogen (BUN) (Figure 4C), serum creatinine (Src) (Figure 4D), and 24 h urinary albumin excretion (Figure 4G) were all significantly higher in five groups of diabetic rats than those in normal group. These values were decreased dose-dependently in icariin treatment groups. Compared with the irbesartan treatment group, the ratio of kidney weight to body weight, serum creatinine, and 24 h urinary albumin excretion were all further decreased, whereas urinary creatinine (Figure 4E) and creatinine clearance (Figure 4F) were increased in icariin (40, 80 mg/kg) groups but these values did not differ between the two groups. Dyslipidemia often occurs in diabetic nephropathy, including increased triglyceride (TG) (Figure 4I), total cholesterol (Figure 4J), non-esterified fatty acid (Figure 4K), low density lipoprotein cholesterol (LDL-C) (Figure 4L) and decreased high density lipoprotein cholesterol (HDL-C) (Figure 4M), these values were dose-dependently improved in icariin (40, 80 mg/kg) groups.

### Icariin Attenuated the Pathological Changes in STZ-Induced Diabetic Rats

**Figure 5:**
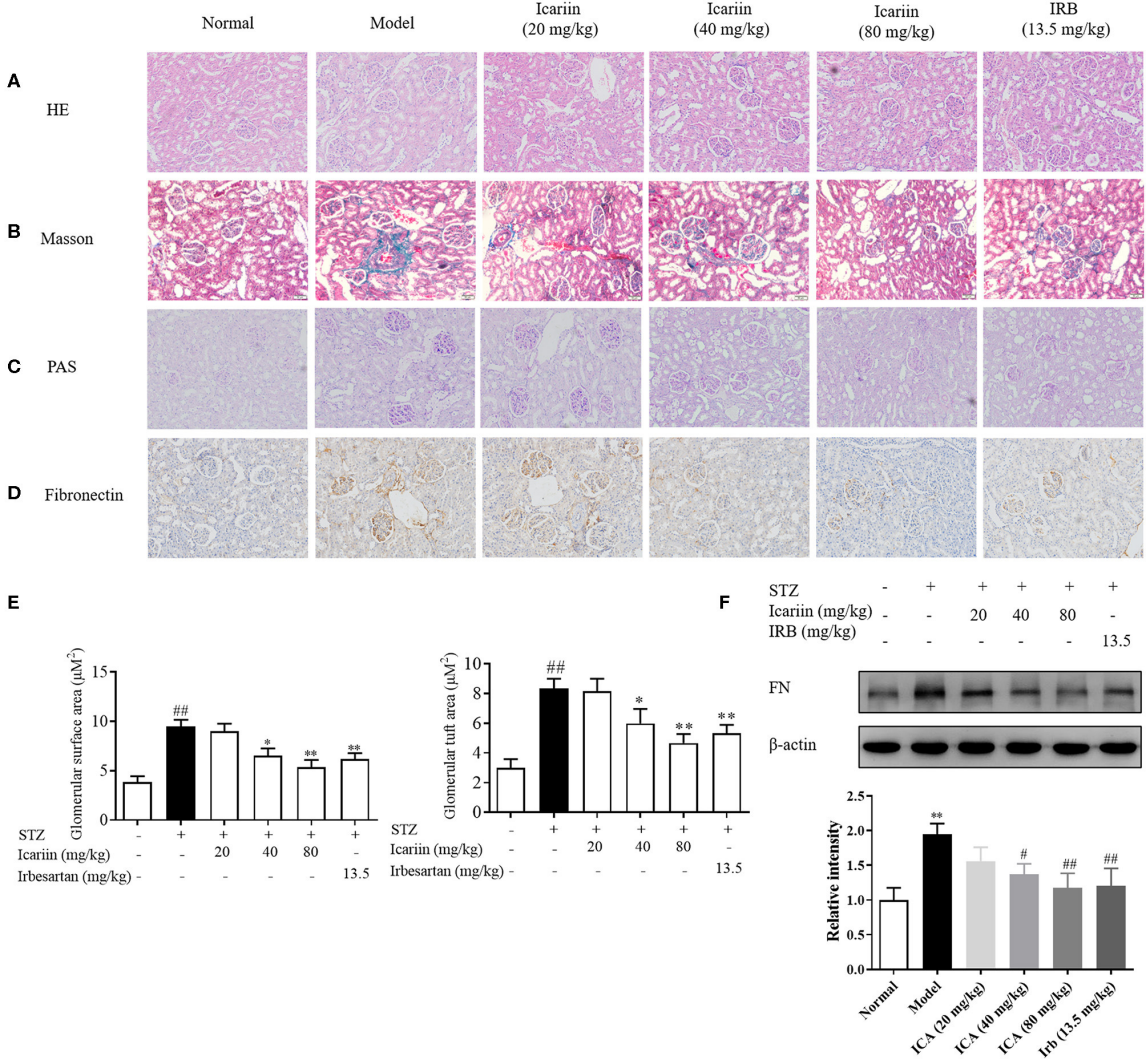
Effect of icariin on the generation of ECM in STZ-induced diabetic rat. **(A)** The histopathology analysis of glomeruli using HE staining (200×). **(B)** The histopathology analysis of glomeruli using PAS staining (200×). **(C)** The histopathology analysis of glomeruli using Masson staining (200×). **(D)** Expression of FN in glomeruli through immunohistochemical (200×). **(E)** Glomerular surface area and glomerular tuft area were quantified using 30 glomeruli per mouse using ImageJ Software. **(F)** Expression of FN protein expression by western blot. Data are expressed as mean ± SD. *p < 0.05, **p < 0.01, compared with the normal control group; ^#^*p* < 0.05, ^*##*^*p* < 0.01, compared with the Model group.

Glomerular lesions are characterized by extracellular matrix deposition in the mesangium and glomerular hypertrophy. In order to observe renal cortical morphological changes in diabetic rats, the representative photomicrographs of rat kidneys in HE, PAS and Masson's trichrome-stained from the six groups were performed. As shown in Figures 5A–C, in model group, the glomerular exhibited alterations in several aspects including extracellular matrix deposition, mesangial region enlargement and basement membrane thickening compared with normal group. Icariin treatment obviously attenuated glomerular hypertrophy and mesangial matrix expansion in diabetic rats. Similarly, the HE staining exhibited increased ECM deposition in the glomeruli of model group, after icariin treatment, the renal injury dose-dependently improved (Figure 5E). Furthermore, the Masson stain also manifested that icariin-decreased collagen deposition in the glomeruli of diabetic rat. Fibronectin (FN), the major component of glomerular ECM, play a key role in promoting glomerular fibrotic lesions. In this study, icariin significantly dampened the increase in FN protein expression in the diabetic rat's kidney by immunohistochemical stain (Figure 5D) and western blot (Figure 5F) assay. Overall, these results showed that icariin alleviated renal damage by reducing the ECM deposition.

### Icariin Attenuated Oxidative Stress in DN Rats and Glomerular Mesangial Cells Induced by High Glucose

**Figure 6:**
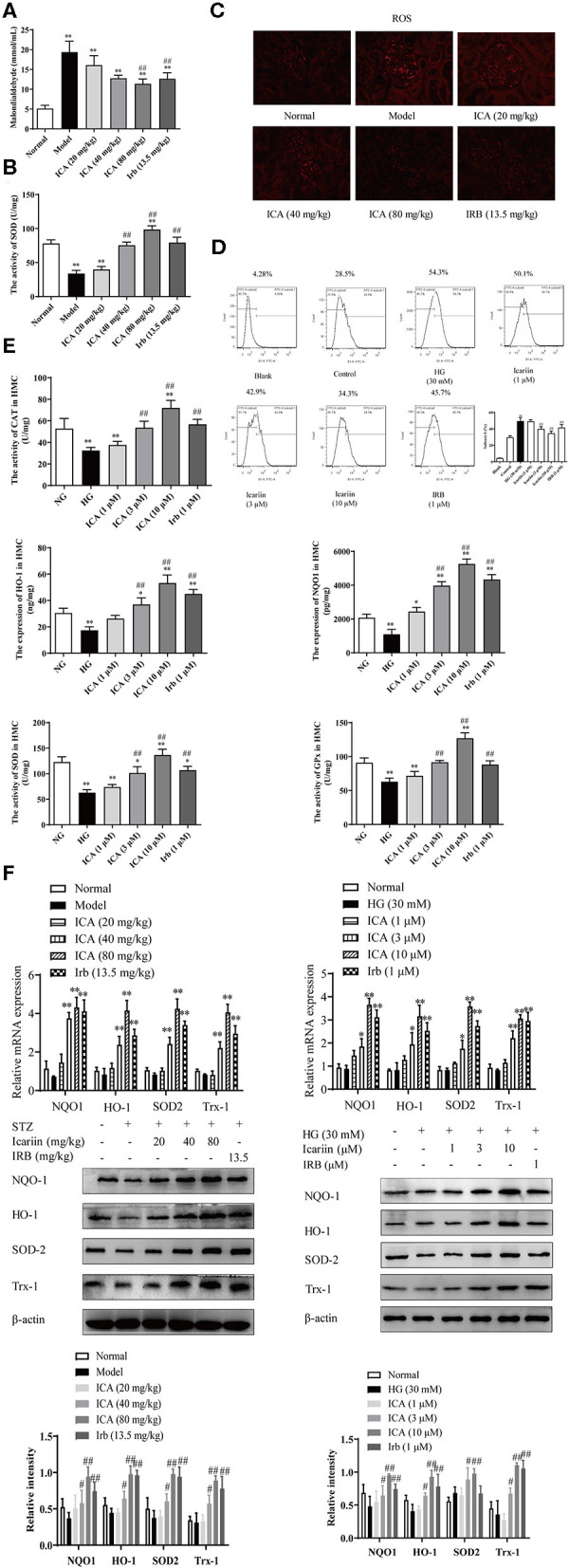
Effect of icariin on oxidative stress in the kidneys of STZ-induced diabetic rats and HMC. **(A)** Malondialdehyde content (MDA). **(B)** Superoxide dismutase (SOD) activity. **(C)** The levels of superoxide anion were detected by dihydroethidium (DHE) staining in kidney. The images were taken at 200× magnification. **(D)** Effect of icariin on the levels of ROS induced by high glucose in HMC and the superoxide anion was detected by flow cytometry. **(E)** Effect of icariin on the activity of antioxidant enzymes CAT, HO-1, NQO1, SOD, and GPx in HMC. **(F)** Effect of icariin on the mRNA and protein level of antioxidant enzymes HO-1, NQO1, SOD and Trx1 in STZ-induced diabetic rats and HMC. Data are expressed as mean ± SD. *p < 0.05, **p < 0.01, compared with the normal control group; ^#^*p* < 0.05, ^*##*^*p* < 0.01, compared with the Model group.

Oxidative stress has been considered to be one of the main causes of excessive generation of ECM and consequent leading to damage to kidney function and structure in DN. To explore the effects of icariin on oxidative stress in DN, the malondialdehyde (MDA) content (Figure 6A), superoxide dismutase (SOD) activity (Figure 6B) and superoxide anion (Figure 6C) as determined by DHE staining in kidney were detected. Compared with normal group, MDA content and superoxide anion were higher, while SOD activity were significantly lower in model group. In contrast, the level of MDA and superoxide anion were markedly lowered, whereas the activity of SOD increased dose-dependently in icariin groups.

The level of ROS in HMC-induced by high glucose, as determined by DHE staining, was markedly increased. The content of superoxide anion (Figure 6D) was dose-dependently attenuated by icariin (1, 3, 10 μM) detected by flow cytometry. During the progression of DN, the antioxidant scavenging system does not work, accompanying by decreased activity and expression of antioxidase, including catalase (CAT), heme oxygenase (HO-1), NADPH: quinone acceptor oxidoreductase 1 (NQO1), superoxide dismutase (SOD) and glutathione peroxidase (GPx). As illustrated in Figure 6E, the stimulation with high glucose significantly decreased the activity of the scavenging system against reactive species in HMC, while icariin promoted the activity of antioxidant enzymes. Moreover, Q-PCR and western blot assays indicated that icariin (1, 3, 10 μM) significantly increased the mRNA and protein expressions of HO-1, NQO1, SOD and Trx1 (Figure 6F) in HMC.

### Icariin-Induced Nrf2 Activation to Inhibit the Oxidative Stress and ECM Generation in DN Rats and Glomerular Mesangial Cells Induced by High Glucose

**Figure 7:**
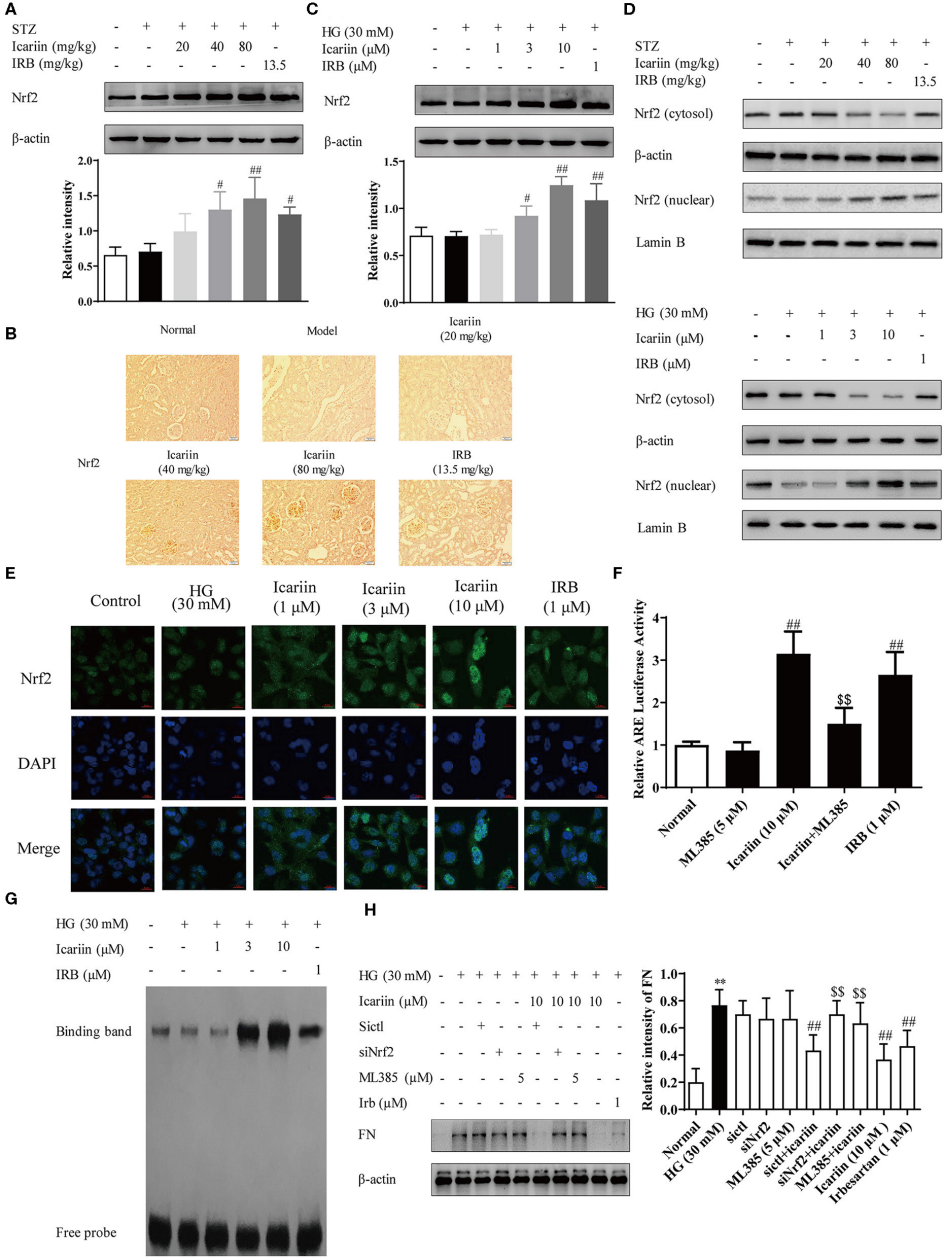
Effect of icariin on Nrf2 activation and ECM generation in DN rats and HMC induced by high glucose. **(A)** Effect of icariin on the total expressions of Nrf2 in DN rats was detected by western blot assy. **(B)** Effect of icariin on Nrf2 protein level in DN rats was detected by immunohistochemical staining. Scale bar: 50 μm **(C)** Effect of icariin on Nrf2 protein level in high glucose induced HMC was detected by western blot assay. **(D)** Effect of icariin on the nuclear expressions of Nrf2 in STZ-induced diabetic rat kidney and high glucose induced HMC was detected by western blot assay. **(E)** Distribution of Nrf2 in HMC through Immunofluorescence. Bar: 20 μm. green fluorescence indicates localization of Nrf2. **(F)** Transcriptional activity of Nrf2 was measured by luciferase reporter assay. **(G)** DNA binding activity of Nrf2 was determined by electrophoretic mobility shift assay. **(H)** Effect of siNrf2 and ML385 on the inhibition of FN by icariin in HMC. Data are expressed as mean ± SD. **p < 0.01, compared with the normal control group; ^#^*p* < 0.05, ^*##*^*p* < 0.01, compared with the Model group or HG group; ^*$$*^*p* < 0.01, compared with the icariin group.

To illuminate the mechanism of icariin on anti-oxidative stress with its effect on the expression and activation of Nrf2, a key regulator of antioxidative stress system was firstly investigated. As shown in the Figures 7A–C, icariin increased the Nrf2 protein level *in vivo* and *in vitro*. Subsequently, we investigated whether icariin played a role in the regulation of Nrf2, and the protein levels of Nrf2 in the nucleus and cytoplasm were detected by western blot assay. The results showed that icariin exerted promotion effects on Nrf2 nuclear translocation compared with the HG group and also in icariin treatment groups of diabetic rats (Figure 7D). To further confirm that icariin promoted HG-induced distribution of Nrf2 in HMC, immunofluorescence staining was performed by laser scanning confocal microscopy to investigate the distribution of Nrf2 (Figure 7E). The images indicated that icariin could significantly increase Nrf2 staining (green) in the nucleus, suggesting that icariin promote the nuclear translocation of Nrf2, and the results were consistent with the western blot. In addition, the DNA binding activity and the transcriptional activity of Nrf2 were detected by EMSA and luciferase reporter gene assay, respectively. As shown in Figures 7F,G, HG stimulation had no effect on the binding activity and luciferase activity of Nrf2 in HMC, while icariin significantly increased the DNA-binding activity of Nrf2 and Nrf2 luciferase activity. The massive deposition of fibronectin (FN) plays an important role in the diabetic glomerular pathology, therefore, inhibition of ECM accumulation in kidneys would repress the glomerulopathy and delay the progression of DN. Figure 7H illustrated that Nrf2 siRNA and ML385 (Nrf2 Inhibitor) also abolished the inhibitory effect of icariin on ECM deposition. Therefore, icariin might alleviate the oxidative stress and ECM generation in HG-induced HMC by activating the Nrf2 signaling pathway.

### Icariin Inhibited HG-Induced Degradation of Nrf2 by Promoting p62-Dependent Keap1 Degradation

**Figure 8:**
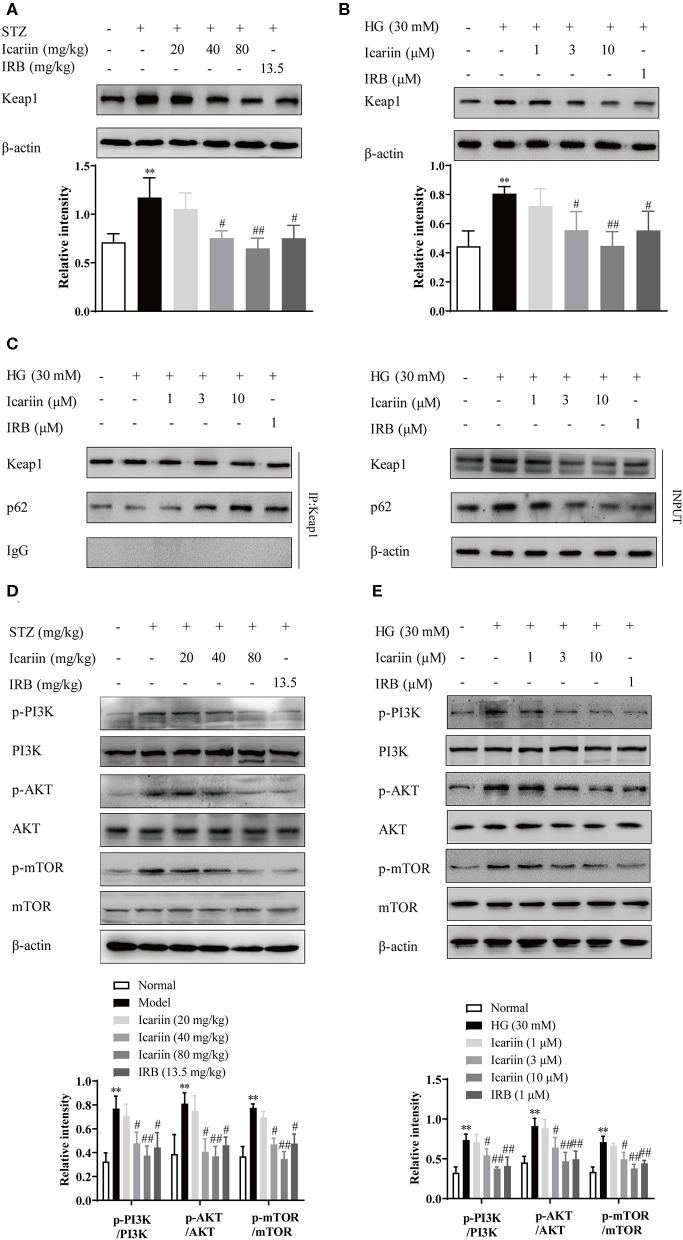
Effect of icariin on HG-induced degradation of Nrf2 by promoting p62-dependent Keap1 degradation. **(A,B)** Effect of icariin on Keap1 protein level in STZ-induced diabetic rat kidney and high glucose induced HMC. **(C)** Effect of icariin on the interaction between p62 and Keap1 induced by high glucose in HMC. **(D,E)** Effect of icariin on phosphorylated and total PI3K, Akt, and mTOR1 protein level *in vivo* and *in vito*. Data are expressed as mean ± SD. **p < 0.01, compared with the normal control group; ^#^*p* < 0.05, ^*##*^*p* < 0.01, compared with the Model group or HG group.

Under normal conditions, the degradation of Keap1 mainly through the proteasomal degradation and autophagy-lysosome pathway (Tkachev et al., 2011). In this process, p62 compete with the Keap1-binding site on Nrf2, subsequently the Keap1-p62 complex is recruited to the autophagosome by LC3, and degraded by autophagy (Fan et al., 2010; Taguchi et al., 2012). To explore the mechanisms by which icariin stimulates Nrf2 activation under high glucose conditions, the protein level of Keap1 was detected by western blot assay (Figures 8A,B). The result showed that Keap1 was significantly increased in model group and HG group as well as it significantly decreased in icariin treatment group. To further confirm that Keap1 is degraded through p62-dependent pathway, the level of co-immunoprecipitated p62 significantly increased after treat with icariin in HMC (Figure 8C), suggesting that p62 was involved in the enhancement effect of icariin on Nrf2 level in HMC under high glucose conditions. In addition, the protein levels of total and phosphorylated PI3K, Akt, and mTOR1 (Figures 8D,E) were examined by western blot assay. Icariin treatment decreased the phosphorylation levels of PI3K, Akt, and mTOR in diabetic rats, these findings revealed that PI3K/Akt/mTOR signal pathway mediated autophagy inhibition is activated by icariin.

### Icariin Reduced the Levels of Superoxide Anion and ECM via the GPER Mediated p62-Dependent Keap1 Degradation and Nrf2 Activation

**Figure 9:**
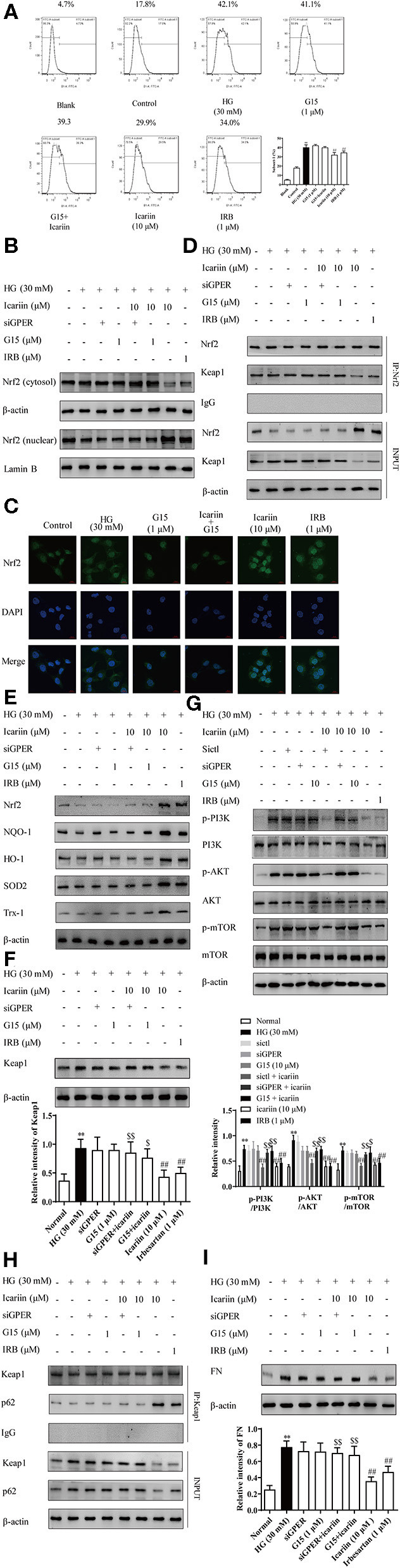
Effect of icariin on GPER mediated p62-dependent Keap1 degradation and Nrf2 activation. The cells were treated with NG (5.6 mM), HG (30 mM), HG (30 mM) + G15 (1 μM), HG (30 mM) + siGPER, HG (30 mM) + icariin (10 μM) + G15 (1 μM), HG (30 mM) + icariin (10 μM) + siGPER, HG (30 mM) + icariin (10 μM) and HG (30 mM) +IRB (1 μM) for 48 h. **(A)** The ROS was detected by flow cytometry. **(B)** the nuclear expressions of Nrf2 induced by high glucose in HMC by western blot. **(C)** Distribution of Nrf2 in HMC through Immunofluorescence **(D)** the interaction between Nrf2 and Keap1 induced by high glucose in HMC. **(E)** The expression of Nrf2 and antioxidant enzymes HO-1, NQO1, SOD2 and Trx1 in HMC. **(F)** Keap1 protein level induced by high glucose in HMC. **(G)** Effect of icariin on phosphorylated and total PI3K, Akt, and mTOR1 protein level induced by high glucose in HMC. **(H)** The interaction between Nrf2 and p62 induced by high glucose in HMC. **(I)** Expression of FN protein expression by western blot. Data are expressed as mean ± SD. **p < 0.01, compared with the normal control group; ^#^*p* < 0.05, ^*##*^*p* < 0.01, compared with the Model group or HG group; ^*$*^*p* < 0.05, ^*$$*^*p* < 0.01, compared with the icariin group.

**Figure 10:**
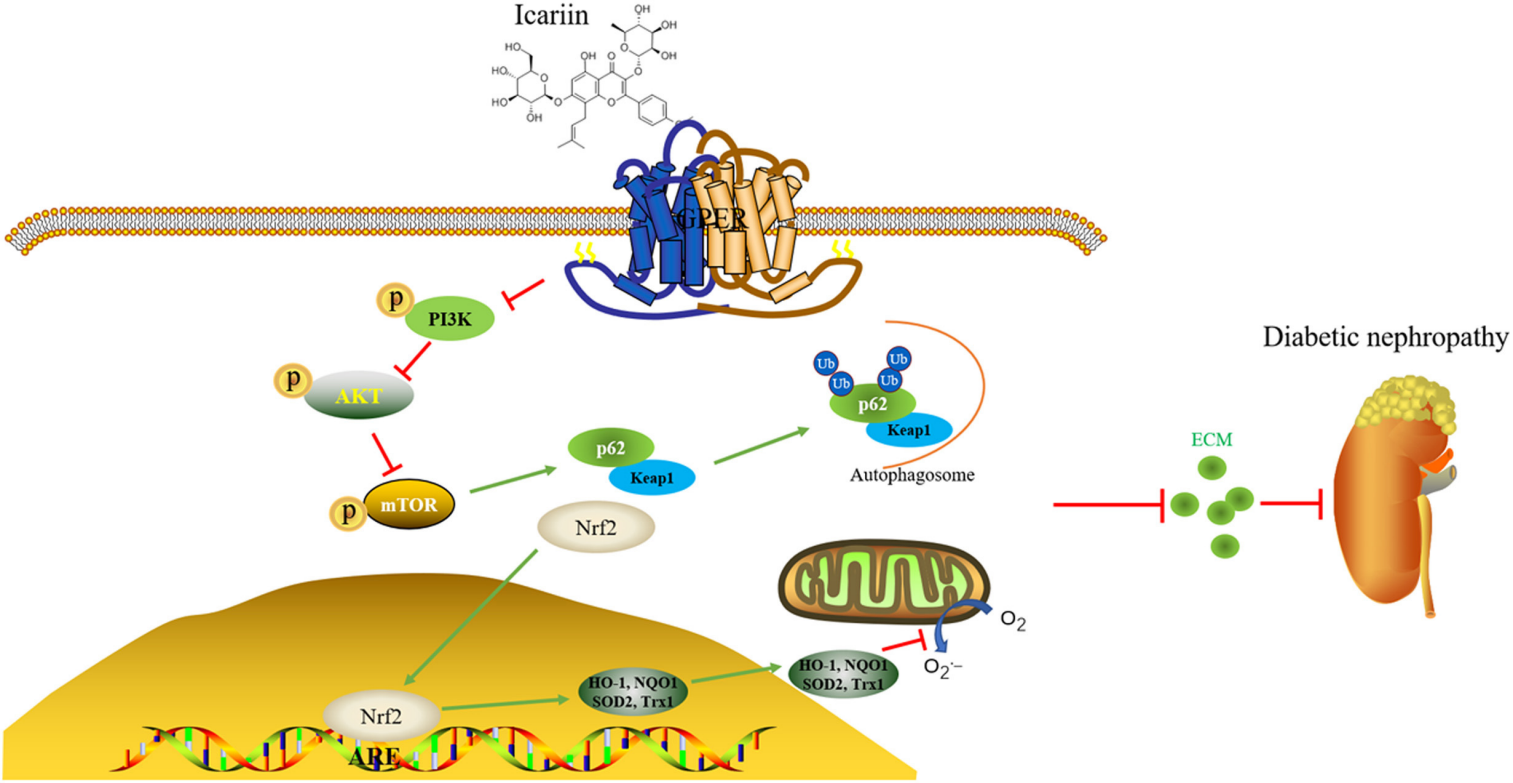
Mechanisms for the anti-diabetic kidney disease (CKD) effect of icariin.

We have previously demonstrated that icariin attenuated high glucose-induced TGF-β production through G protein-coupled estrogen receptor 1 (GPER) (Li et al., 2013). Here, we additionally demonstrated significant increases in ROS accumulation as indices of oxidative stress during the progression of DN, and explored associated mechanisms. To confirm the key role of GPER played in icariin mediated protection of DN, the level of ROS (Figure 9A) was detected by flow cytometry. The results showed that icariin markedly reduced the superoxide anion in HMC, while G15 (a selective antagonist of GPER) prevented icariin decreasing ROS. Then, the protein levels of Nrf2 in the nucleus and cytoplasm (Figures 9B,C), the interaction between Nrf2 and Keap1 (Figure 9D), the antioxidant enzymes (Figure 9E), Keap1 (Figure 9F), PI3K/Akt pathway (Figure 9G), the interaction between p62 and Keap1 (Figure 9H), and FN (Figure 9I) were detected. siGPER and G15 significantly reduced the inhibitory effects of icariin on Keap1, PI3K/Akt pathway, p62 and FN expression. In addition, icariin also significantly up-regulated the Nrf2 and the antioxidant enzymes protein level, increased the dissociation of Nrf2 and Keap1, association of Nrf2 and p62 in HMC, while these effects could be abolished by siGPER and G15. The main mechanisms about anti-CKD effect of icariin has been showed in (Figure 10).

## Discussion

Diabetic kidney disease (DKD) is a major cause of mortality worldwide in diabetic, and decreased kidney function can be formed by many different reasons including hypertensive nephrosclerosis and acute kidney failure (Groop et al., 2009; Gregg et al., 2014). Diabetic nephropathy (DN) is a diagnosis that indicates functional changes and specific pathologic structural in the kidneys of type 1 or type 2 DM patients. These changes would lead to clinical manifestations which characterized by proteinuria, hypertension, and gradual decline in kidney function (Umanath and Lewis, 2018). Multiple mechanisms have been proposed to mediate adverse hyperglycemic effects, including the increased production of sorbitol, methylglyoxal, diacylglycerol, reactive oxygen species, and advanced glycation end products (Lee et al., 1989; Giacco et al., 2014). However, the precise pathogenesis of DN remains unclear.

Previous studies suggested that icariin could be beneficial to the prevention of DN due to its ability to reduce serum creatinine (Cr), blood urea nitrogen (BUN), TGF-β1 protein expression in STZ-induced diabetic rats (Qi et al., 2011), and block TGF-β1-Smads pathway of mesangial cells induced by high glucose, which is beneficial to the prevention of DN (Li et al., 2013). Unlike previous studies, our present work mainly explore the therapeutic effect of icariin on DN and its underlying mechanism through focusing on oxidative stress. In this study, a rat model of diabetes was induced by STZ, a method which has been widely used to create diabetes models in rodents with pathological features similar to human diabetes (Tesch and Allen, 2007). One week after STZ injection, blood glucose was assessed with the result that about 94 % of female rats and 85% of male rats developed diabetes. It has been reported that the evaluation of drugs' effects on diabetic nephropathy should not be examined until 3 weeks after STZ injection when the kidney gets out of the acute mild nephrotoxic (Kraynak et al., 1995). Therefore, the diabetic nephropathy model, as exhibited by weight loss, food intake, water intake, blood glucose, 24 h urine volume, 24 h proteinuria, blood urea nitrogen (BUN), serum creatinine (Src), urine creatinine (Ucr), and blood pressure in Figure 2, had been evaluated at 4 weeks after intraperitoneal injection of STZ. The result showed that about 92 % of female rats and 82% of male rats developed diabetes nephropathy. Compared with normal rats, diabetic rats showed a significant increase in food and water intake, blood glucose, 24 h urine volume, 24 h proteinuria, blood urea nitrogen (BUN), serum creatinine (Src) and blood pressure with the reduction of urine creatinine (Ucr) and weight.

After the evaluation, the healthy rats and successfully-established DN rats were divided into six groups. Body weight, food intake, water intake and blood glucose were recorded and the effects of icariin on renal function, as exhibited by 24 h urine volume, 24 h proteinuria, urine creatinine (Ucr), were measured every 3 weeks during the 9-weeks experimental period. Results showed that icariin (40, 80 mg/kg) could decrease the 24 h urine volume and 24 h proteinuria with urine creatinine (Ucr) increased right from the first detection after 3-weeks administration (date not shown). Then, we demonstrated that icariin reduced DN progression in a dose-dependent manner, as exhibited by reducing weight loss, water intake, food intake and blood pressure during the process of 9-weeks administration. Diabetic rats presented with severe renal dysfunction manifested as increased urine volume in 24 h, proteinuria in 24 h, blood urea nitrogen (BUN), serum creatinine (Src), urinary albumin excretion in 24 h and the ratio of kidney weight to body weight, as well as urinary creatinine and creatinine clearance decreased. These values were decreased in a dose-dependent manner in all treatment groups, whereas the urinary creatinine and creatinine clearance rate were markedly increased only in icariin (40, 80 mg/kg) treatment groups. In addition, icariin treatment markedly attenuated glomerular hypertrophy, mesangial matrix expansion and the renal damage, as well as collagen deposition in the glomeruli of diabetic rats decreased. These results reported the therapeutic effects of icariin on DN rats by attenuating glomerular hyperfiltration, kidney structure deterioration and decreasing collagen deposition. Furthermore, we found that the treatment of icariin and irbesartan had no effect on STZ-induced hyperglycemia in type 1 diabetic rats, suggesting that the renal protective effects of icariin and irbesartan were probably independent of blood glucose control, providing support for glycemic control and lent support to previous studies (Cheng et al., 2001; Shao et al., 2008).

Typical pathological features of DN are glomerular hypertrophy and mesangial matrix expansion. We found that ECM was significantly increased in model group as compared with rats in normal group. There are complicated mechanisms in the increased ECM of the glomeruli, including hemodynamic change, lipid and glucose metabolisms abnormalities, multiple cytokines and inflammatory factors along with oxidative stress (Kanwar et al., 2011; Jha et al., 2017). Oxidative stress is a crucial pathogenic factor of vascular complications in metabolic disease including diabetic nephropathy, which leads to the development and progression of kidney injury (Forbes et al., 2008; Sharma, 2016). Mesangial cells generated a mass of ROS induced by high glucose, which might promote the ECM deposition by promoting the production of plasminogen activator inhibitor (Zheng et al., 2011), activating TGF-β1-Smad signaling pathway (Chen et al., 2019) and the protein kinase C pathway (Ji et al., 2016). In the present study, icariin inhibited the expression of FN, the main component of ECM, in HMC induced by high glucose. The levels of ROS are regulated by the balance of endogenous antioxidants and anti-oxidases, which indirectly reflect the ability to eliminate ROS (Cao et al., 2016). MDA, an end-product of lipid peroxidation, directly reflect the extent of lipid peroxidation (Huang et al., 2013). At the same time, icariin treatment restored SOD activities, decreased MDA and ROS levels compared with model group. In addition, icariin increased the activities and promoted the protein levels of antioxidant enzymes, thus inhibiting the oxidative stress and reducing the intracellular levels of ROS in cells. These results indicated that icariin could alleviate oxidative stress in diabetic rats and mesangial cells induced by high glucose.

Nrf2, as a redox-sensitive transcription factor, is a key transcription factor that regulates the expression of various antioxidant enzymes to maintain cellular redox homeostasis and prevent oxidative stress (Saito, 2013; Suzuki and Yamamoto, 2017). In the rest time, Nrf2 is anchored in the cytoplasm. In the presence of external stimuli, Nrf2 accumulates and transports to the nucleus, and then interacts with antioxidant-response element (ARE) to initiate the response (Kaspar et al., 2009). Sulforaphane, a Nrf2 activator, could reduce renal damage and protect renal function in diabetic mice, suggesting that Nrf2 might be a promising therapeutic target for DN (Zheng et al., 2011). In addition, there is evidence that Nrf2 knockout mice or silencing of Nrf2 in renal cells might increase the accumulation of ECM deposition in diabetes (de Haan, 2011). In this study, icariin and irbesartan were found to promote the total protein levels and nuclear translocation of Nrf2, thus promoting the protein expression of HMC antioxidant enzymes under high glucose treatment. Herein, the studies showed that nuclear translocation, DNA binding activity and transcriptional activity of Nrf2 decreased and FN protein level increased in HG-induced HMC. Moreover, further studies confirmed that icariin upregulated of nuclear Nrf2 DNA binding activity and Nrf2 transcription activity in HG-induced HMC. In combination with siNrf2 and ML385, icariin mediated reduction of FN was almost abolished, which meant that icariin needed to active Nrf2 to inhibit the oxidative stress and ECM production of DN rats and mesangial cells induced by high glucose.

In response to oxidative stress, electrophilic damage targets the Nrf2-Keap1 complex, separates Nrf2 from Keap1, enable Nrf2 to translocate into the nucleus to transactivate target genes and facilitates the degradation of Keap1 (Suzuki and Yamamoto, 2017). The degradation of Keap1 is mainly goes through ubiquitin-proteasome and autophagy-lysosome pathway (Tkachev et al., 2011). P62, the selective autophagy substrate and the protein levels are generally considered to be inversely proportional to autophagy activity, keeps a binding site for Keap 1 and promotes autophagic degradation of Keap1, releasing Nrf2, and transporting Nrf2 into the nucleus to transcript antioxidant genes to prevent oxidative stress (Komatsu et al., 2010). Indeed, our study provided direct evidence to demonstratd that icariin would reduce the protein level of keap1 and p62 but increased the correlation level between p62 and Keap1 and subsequent degradation level of Keap1, indicating that icariin was found significantly, but not completely, to prevent the diabetes-induced up regulation of Keap1. Impaired autophagy activity contributes to the pathogenesis of DN, and the recovery of autophagy activity may be a promising therapeutic target for DN. Currently, the latest studies have confirmed that the inhibition of PI3K/Akt/mTOR signaling pathway restored autophagy and ameliorate DN (Tu et al., 2019; Wang et al., 2019). Result showed that the phosphorylation levels of PI3K, Akt, and mTOR in diabetic rats and mesangial cells induced by high glucose were obviously decreased by icariin treatment.

GPER has been shown to play a part in the rapid non-genomic signaling events of E2 widely observed in cells and tissues (Revankar et al., 2005). Using isolated perfused hearts subjected to ischemia/reperfusion, pretreatment with the GPER agonist, G1, confers cardioprotective effects via PI3K pathways (Bopassa et al., 2010). In order to confirm the crucial role of GPER in the *in vivo* and *in vitro* protection of DN-mediated by icariin, combined use with G15 demonstrated the involvement of GPER in the inhibition of icariin on ROS generation. These findings suggested that GPER might involve in the regulation of icariin on intracellular homeostasis. Further studies demonstrated that siGPER or G15 could eliminate the promotion of icariin on the antioxidant enzymes protein level and the dissociation of Nrf2 and Keap1, indicating that icariin inhibited the degradation of Nrf2 depend on GPER in HMC. Moreover, the combined use of siGPER and G15 nearly eliminated the attenuation of icariin on the association of Keap 1 with p62 and ECM generation in HMC, illustrating that Nrf2 pathway GPER was also important in the icariin inhibition of the oxidative stress and the ECM generation in high glucose-stimulated HMC.

## Conclusion

Overall, icariin, a potential activator of GPER, reduced the levels of ROS and ECM generation in the kidney of diabetic rats, and ameliorated the symptoms of DN through, at least partially, GPER mediated p62-dependent Keap1 degradation and Nrf2 activation.

## Data Availability Statement

The datasets generated for this study are available on request to the corresponding author.

## Ethics Statement

The animal study was reviewed and approved by the Animal Ethics Committee of China Pharmaceutical University.

## Author Contributions

XD and KW designed the study. KW, XZ, ZP, WY, and XW performed experiments. XD and KW contributed significantly to analysis and manuscript preparation. KW, XZ, and XG wrote the manuscript. All authors contributed to the article and approved the submitted version.

## Conflict of Interest

The authors declare that the research was conducted in the absence of any commercial or financial relationships that could be construed as a potential conflict of interest.
